# The Relationship Between Psychological Distress and Physical Activity Is Non-linear and Differs by Domain: a Cross-Sectional Study

**DOI:** 10.1007/s12529-022-10130-5

**Published:** 2022-09-30

**Authors:** David Mizrahi, Christopher T. V. Swain, Fiona Bruinsma, Allison Hodge, Natalie Taylor, Brigid M. Lynch

**Affiliations:** 1 The Daffodil Centre, The University of Sydney, a joint venture with Cancer Council NSW, Sydney, Australia; 2 Prince of Wales Clinical School, UNSW Sydney, Sydney, Australia; 3 Cancer Epidemiology Division, Cancer Council Victoria, Melbourne, Australia; 4 Centre for Epidemiology and Biostatistics, Melbourne School of Population and Global Health, The University of Melbourne, Melbourne, Australia; 5 School of Population Health, UNSW Sydney, Sydney, Australia; 6 Physical Activity Laboratory, Baker Heart and Diabetes Institute, Melbourne, Australia

**Keywords:** Exercise, Physical activity, Sedentary, Mental health, Psychological distress

## Abstract

**Background:**

There is increasing evidence for the relationship between physical activity (PA), sedentary behaviour and mental health. Limited data exists on sex-specific associations. We aimed to identify associations between PA dose and domain and television time with psychological distress, including sex-stratified models.

**Methods:**

A total of 22,176 adults from the Melbourne Collaborative Cohort Study follow-up 2 cohort (2003–2007) participated in this cross-sectional study. Occupational, household, transport, leisure PA, hours watching television and psychological distress were assessed. Restricted cubic splines were used to examine the relationships between PA domains, television viewing time and psychological distress.

**Results:**

The relationships between PA and psychological distress were non-linear (*p* < 0.05) and differed by PA domain. There were dose-dependent, inverse associations between distress with transport (*B*[95% CI] = −0.39[−0.49, −0.30]) and leisure PA (*B*[95% CI] = −0.35[−0.46, −0.25]). The effect estimates for transport and leisure PA with distress were larger for women. For household domain, a U-shaped curve with an elongated tail was seen. Median PA was associated with lower distress compared with lower quantities (*B*[95% CI] = −0.12[−0.22, −0.03]); however, this association was not evident with increasing household PA. There were no clear associations between occupational PA and distress. Higher television viewing was associated with higher distress (*B*[95% CI] = 0.16[0.02, 0.30]).

**Conclusions:**

Increasing PA and reducing television viewing may contribute to reduced psychological distress, particularly in women. Future interventions should incorporate leisure and transport PA and decrease television viewing to assess the impact on mental health.

**Supplementary Information:**

The online version contains supplementary material available at 10.1007/s12529-022-10130-5.

## Introduction

Reduced mental health is an increasing burden in our society, which contributes globally to increases in paid sick leave, job dissatisfaction and increasing healthcare costs [1, 2]. Reduced mental well-being can be difficult to manage due to vagaries around diagnostic criteria, best-practice management and support in reporting symptoms [3].

Physical and mental health appear to be intricately linked. Just as low physical activity (PA) can predict the development of a mental health disorder [4], people with diagnosed mental health conditions often present with reduced PA and physical fitness [5]. Given this, PA is now promoted as a tool for the prevention and management of mental health conditions and has demonstrated the ability to improve mental health in a numerous populations [6]. Mechanistically, PA produces neurobiological responses including the upregulation of brain-derived neurotrophic factor and activating the endocannabinoid system, both of which contribute to the anti-depressant effects of exercise [7], with a common example termed the ‘runner's high’ [8]. However, it is unclear how the setting where PA is performed can impact mental health outcomes such as psychological distress.

Evidence from observational studies and randomized controlled trials using tightly designed exercise interventions generally focus on the leisure PA domain, which includes structured exercise, sports or recreational activities. This research typically concludes that leisure PA improves overall mental health [6, 9]. Increasing psychological distress over time has been shown to be associated with reducing moderate-to-vigorous PA [10]. Less is known about how PA accumulated in other domains is related to psychological distress, including transport (e.g. purposefully walking to destinations), household (e.g. gardening, cleaning) and occupational PA (e.g. light office work, heavy manual labour). A recent meta-analysis suggested that the relationship between PA and mental health is dependent on the domain the PA is accrued, with greater associations among leisure and transport PA and with less clear associations among occupation and household PA [11]. However, this review identified a limited number of studies examining occupation, household or transport domains; did not examine sedentary behaviours such as television viewing time; did not explore dose–response relationships; and was marked by high, unexplained heterogeneity [11].

Sedentary behaviours such as television time have been associated with psychological distress [12]. Binge television viewing can reduce sleep quality, which can then contribute to increased distress [13]. Conversely, exposure to distressing television content has been shown to potentially reduce sources of distress by raising awareness, reducing stigma and shifting attitudes in real-life scenarios [14]. High amounts of television viewing is associated with increased BMI, cholesterol, blood pressure and triglycerides [15], and when combined with low PA is associated with earlier mortality [16]. Nearly two-thirds of American adults and children spend ≥ 2 h/day watching television, displaying increased daily sitting time compared with prior decades [17]. Additionally, television viewing could have a bidirectional relationship with mental health outcomes [18].

The World Health Organization recommends adults reduce sedentary behaviours and encourages movement across all PA domains [19]. Yet, physiological and psychological responses to PA may differ by the domain in which it is performed. PA can be performed as recreational (e.g. organized sport, hiking), lifestyle (e.g. gym classes, cycling to work) or essential activities (e.g. gardening, housework, physical work tasks). Critically, females have been shown to have lower levels of physical activity [20] and higher rates of psychological disorders than males [21]. Thus, sex-specific research on this relationship is required to bridge these gaps and identify whether sex-specific interventions are needed.

The aim of this study was to examine dose–response associations between domains of PA (leisure, household, transport and occupational) and television viewing time with psychological distress, including sex-specific associations. We hypothesized that higher PA and less television viewing time would be associated with reduced psychological distress. We also hypothesized that associations between PA and psychological distress would be stronger for leisure and transport PA than for occupational and household PA.

## Materials and Methods

This study utilized data from the Melbourne Collaborative Cohort Study (MCCS) [22], which is a prospective study undertaken to investigate relationships between socio-demographic factors, lifestyle patterns and chronic disease risk. In brief, 24,469 female and 17,044 male adults were recruited from the Melbourne metropolitan area (1990–1994). As baseline data on PA were not domain-specific and did not contain information on duration of PA or its intensity, this study uses exposure and outcome data from follow-up 2 (FUP2, 2003–2007). We excluded participants who were missing data for both PA and television viewing time, mental health outcomes and confounders. The study protocol was approved by Cancer Council Victoria’s Human Research Ethics Committee, and all participants provided written informed consent [22].

### Physical Activity and Television Viewing Time Assessment

Self-reported PA and television viewing time were collected at FUP2 by trained interviewers. For PA, a modified version of the long-form International Physical Activity Questionnaire (IPAQ-long) was used to obtain information on occupational, home (including household and garden work), transport and leisure PA completed in the previous 3 months [23], with Cronbach’s *α* = 0.63–0.85 [24]. For the household, transport and leisure domains, metabolic equivalents of tasks (METs) were calculated by multiplying hours per week by the intensity level assigned by the IPAQ-long guidelines. For the occupational domain, participants were additionally asked to select their usual occupational PA intensity level from a 4-point ordinal scale (1 = ‘Mainly sitting’, 4 = ‘Hard physical effort, e.g. scrubbing floors, digging, heavy lifting’), with the Compendium of Physical Activities used to assign a MET value [25]. ‘Mainly sitting’ = 1.5 METs; ‘Mainly sitting with occasional walking and moving about to do tasks’ = 1.87 METs; ‘Mainly on feet with some light carrying or lifting’ = 3.0 METs; and ‘Hard physical effort’ = 6.5 METs. Only participants who indicated they were currently working or volunteering provided data for occupational PA. Instead of asking about total sitting time, participants reported the total time spent watching television on mid-week and weekend days, with the average number of hours/day calculated. Television viewing time has been shown to correlate fairly with accelerometer-assessed sedentary time [26]. During FUP2 of the MCCS, watching television was the most commonly reported leisure-time activity amongst Australian adults [16].

### Psychological Distress Assessment

At FUP2, mental health was assessed using the Kessler Psychological Distress Scale (K-10). The K-10 measures psychological distress over the previous 4 weeks, and is scored based on responses to 10 items, which are summed to give a total score between 10 and 50. Scores of 20–24, 25–29 and 30–50 reflect mild, moderate and severe psychological distress, respectively [27]. It has been identified as a valid, reliable and sensitive measure of mental health, is used clinically in Australia and is suitable for epidemiological research [28], with Cronbach’s *α* > 0.88 [29].

### Confounders

We constructed a directed acyclic graph (DAG, Supplement 1A) to guide our selection of confounders for adjustment. These included age (years), sex, country of birth (Australia/New Zealand, northern Europe, southern Europe), education (primary school, some high school, completed high school, tertiary education), marital status (married/de facto, single, separated/divorced, widowed), socio-economic index of areas (SEIFA) quintiles, alcohol consumption (lifetime abstainer, former drinker, drinker), smoking status (never smoker, former smoker, smoker), comorbidities (yes/no) and working status (some paid or volunteer work/not working). As PA and sedentary time can affect body composition, we did not include body composition as a confounder in our main analysis. However, as people with higher body composition may be less likely to participate in PA, body mass index (BMI, kg/m^2^) was included as a confounder in a sensitivity analysis (Supplement 1B). To reflect temporal sequencing and to ensure these were confounders, we used baseline data for confounders, except age and working status, which were collected at FUP2.

### Statistical Analysis

Participant data were presented using frequencies and percentages for categorical variables and means and standard deviation (SD) or medians and interquartile ranges (IQR) for continuous variables, depending on normality of distribution. Following the methods outlined by Desquilbet and Mariotti [30], restricted cubic splines were used to graphically represent the dose–response association between PA domains and television viewing time with psychological distress, to test whether these associations were linear, and to generate effect estimates for each exposure–outcome association [30]. For occupational, household and transport PA, and television viewing time, four knots were placed at the 5th, 35th, 65th and 95th percentiles. For leisure PA, as the 50th percentile was not different to the 5th percentile, knots were placed approximately at the 5th, 60th, 80th and 95th percentiles. To assess whether associations were linear, linear models that included continuous measures of each domain were compared with models that contained the restricted cubic splines using the likelihood ratio test [31]. We then fitted a linear regression model using the spline transformed exposures. The reference level for each model was set at the 25th percentile for each exposure, and each model was adjusted for the confounders outlined above. Analyses were performed for the entire sample and stratified by sex. All analyses were performed using Stata version 14.2 (Stata Corporation, College Station, TX, USA).

## Results

**Fig. 1:**
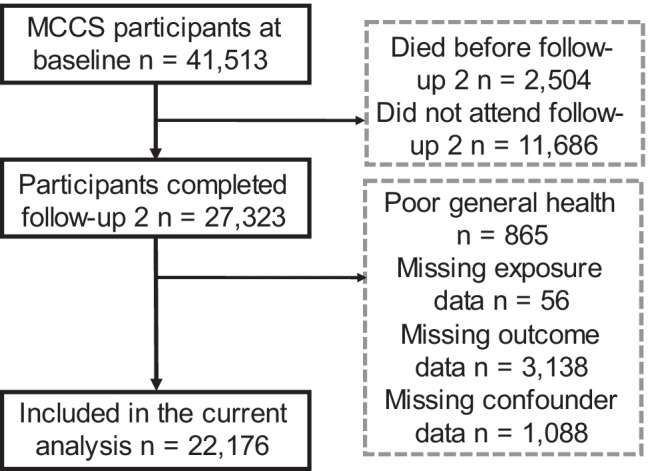
Recruitment flow chart

Participant selection is displayed in Fig. 1, and participant descriptive data is provided in Table 1. The study sample consisted of 22,176 participants who were aged between 48 and 87 years. Television viewing time data was available for 19,310 participants, whilst occupational PA data was only available in participants who were employed at FUP2 (*n* = 8955).

**Table 1:** Participant descriptive characteristics

Age, *years*, mean (SD)	65.8 (8.7)
Sex, *n* (%)	
Female	13,382 (60)
BMI, kg/m^2^, mean (SD)	26.4 (4.2)
Country of birth, *n* (%)	
Australia/New Zealand	17,138 (77)
Northern Europe	1615 (7)
Southern Europe	3423 (15)
Education, *n* (%)	
Primary school	2359 (11)
Some high school/technical school	8568 (39)
Completed high school/technical school	2336 (10)
Tertiary/diploma/degree	8913 (40)
Baseline marital status, *n* (%)	
Married/de facto	16,463 (74)
Single	2055 (9)
Separated/divorced	2273 (10)
Widowed	1385 (6)
Baseline SEIFA, *n* (%)	
1^st^ quintile	3217 (15)
2^nd^ quintile	3920 (18)
3^rd^ quintile	3417 (15)
4^th^ quintile	4507 (20)
5^th^ quintile	7115 (32)
Baseline drinking status, *n* (%)	
Lifetime abstainer	5541 (25)
Former drinker	2243 (10)
Drinker	14,392 (65)
Baseline smoking status, *n* (%)	
Never smoked	13,421 (40)
Former smoker	6846 (31)
Smoker	1909 (9)
Baseline comorbidities, *n* (%)	
Cardiometabolic comorbidities	4688 (21)
Arthritis	6377 (29)
Physical activity, *n* (%)	
Inactive	1417 (7)
Insufficiently active	4837 (22)
Sufficiently active	15,493 (71)
Kessler Psychological Distress Scale, *n* (%)	
Likely to be well	19,538 (88)
Likely to have a mild disorder	1636 (7)
Likely to have a moderate disorder	623 (3)
Likely to have a severe disorder	379 (2)

Data are presented as mean (SD) for continuous variables (age, BMI, mental health score), and *n* (%) for categorical variables (sex, education, socio-economic index, employment, smoking status, alcohol intake, physical activity and comorbidities)

*BMI* body mass index, *SEIFA* socio-economic index of areas

The median (IQR) quantity of PA was 67 (30, 94) MET h/week for occupational PA, 22 (8, 44) MET h/week for household PA, 10 (4, 20) MET h/week for transport PA and 0 (0, 12) MET h/week for leisure PA (55% of participants reported no leisure PA). Participants reported watching a median (IQR) 2.4 (1.6, 3.4) h of television per day.

The total scores for psychological distress (K-10) are presented in Table 1, with responses to individual items presented in Table 2. Using established scoring methods for the K-10 in Australian adults [32], 88% of participants could be classified as likely to be well, 7% as likely to have a mild mental disorder, 3% as likely to have moderate mental disorder and 2% as likely to have a severe mental disorder.

**Table 2:** Frequency and percentage of responses to individual psychological distress items

	None of the time	A little of the time	Some of the time	Most of time	All of the time
	*n* (%)				
Felt tired for no reason	9155 (41)	7095 (32)	4475 (20)	1209 (6)	242 (1)
Felt nervous	10,838 (49)	7531 (34)	2998 (14)	601 (3)	208 (1)
So nervous, could not be calmed	20,090 (91)	1458 (7)	438 (2)	123 (1)	67 (0.3)
Felt hopeless	18,246 (82)	2831 (13)	843 (4)	181 (1)	75 (0.3)
Felt restless or fidgety	12,680 (57)	7058 (32)	2034 (9)	313 (1)	91 (0.4)
So restless, could not sit still	18,337 (83)	2726 (12)	826 (4)	193 (1)	94 (0.4)
Felt depressed	13,697 (62)	6064 (27)	1902 (9)	395 (2)	118 (1)
Felt everything an effort	12,480 (56)	6760 (31)	2128 (10)	639 (3)	169 (1)
So sad, could not cheer up	18,679 (84)	2489 (11)	723 (3)	210 (1)	75 (0.3)
Felt worthless	19,085 (86)	2192 (10)	617 (3)	193 (1)	89 (0.4)

**Fig. 2:**
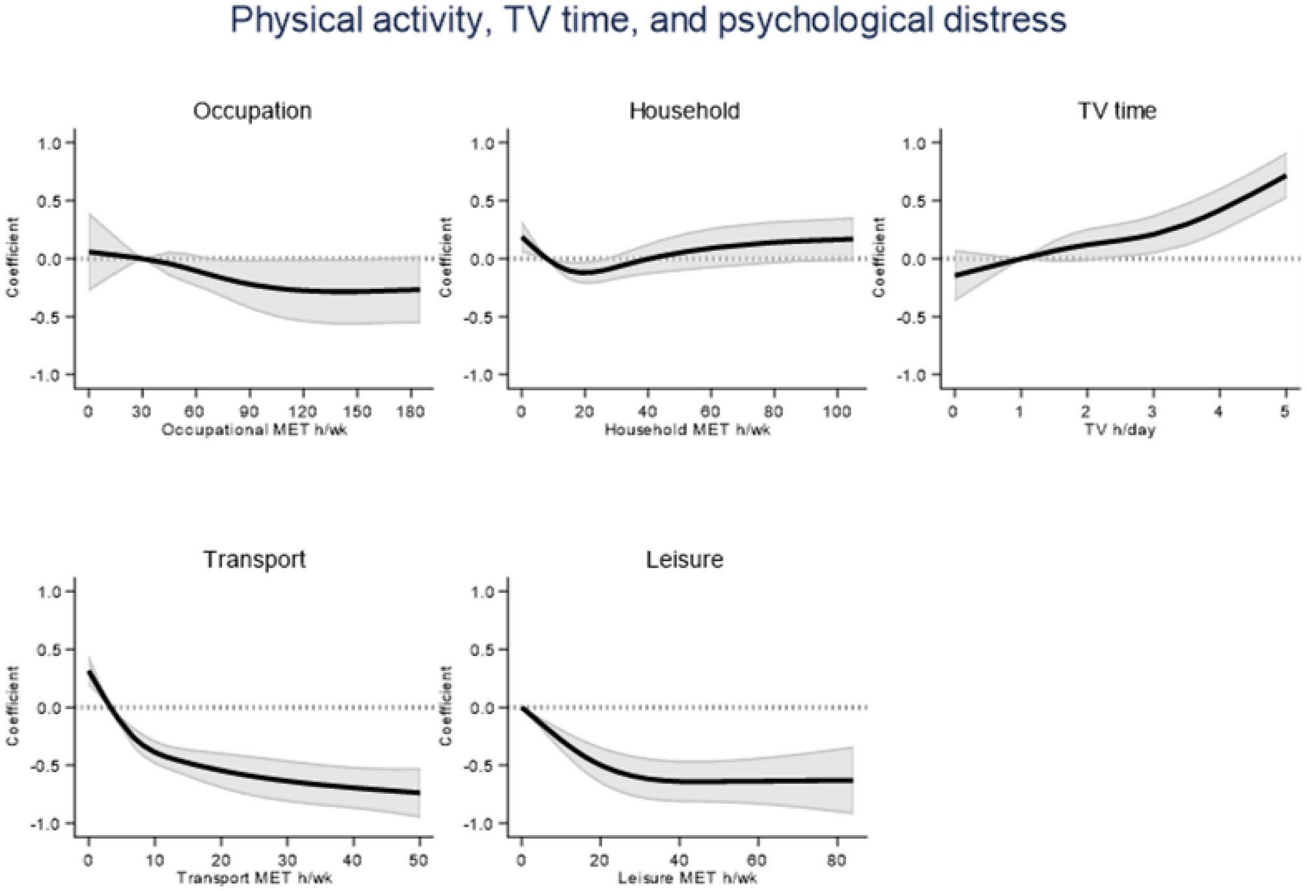
Dose–response relations for physical activity domains and television viewing time with psychological distress measured with the K-10. The solid line represents the regression coefficient, and the shaded area represents the 95% confidence interval. Higher transport and leisure physical activity were associated with less psychological distress. There were no clear associations for occupational or household physical activity. Higher levels of television viewing time were associated with more psychological distress. Associations were adjusted for age, sex, country of birth, education, marital status, socio-economic index of areas, alcohol consumption, smoking status, comorbidities and working status

**Fig. 3:**
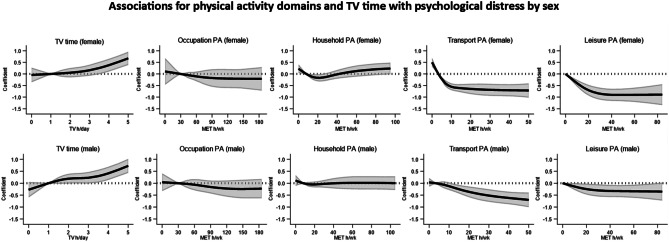
Sex-specific dose–response relationship for physical activity domains and television viewing time with psychological distress measured with the K-10. The solid line represents the regression coefficient, and the shaded area represents the 95% confidence interval. Associations were adjusted for age, sex, country of birth, education, marital status, socio-economic index of areas, alcohol consumption, smoking status, comorbidities and working status

Associations between PA domains, television viewing time and K-10 scores were non-linear (*p* < 0.05) and are depicted in Fig. 2. Higher transport and leisure PA was associated with lower psychological distress, suggesting better overall mental health. Specifically, compared to participants in the 25th percentile, median transport PA was associated with lower K-10 scores (*B*[95% CI] = −0.39[−0.49, −0.30]). For leisure PA, compared to participants reporting no PA, participants in the 75th percentile reported lower K-10 scores (*B*[95% CI] = −0.35[− 0.46, − 0.25]). Likewise, median household PA was associated with improved K-10 scores (*B*[95% CI] = − 0.12[−0.22, −0.03]), but these associations were not evident for higher levels of household PA, with a U-shaped curve and an elongated tail seen. There were no clear associations between occupational PA and distress. Specifically, participants who reported median occupational PA reported similar K-10 scores (*B*[95% CI] = −0.13[−0.28, 0.01]) as participants in the 25th percentile. More television viewing time was associated with higher levels of psychological distress. Compared to the 25th percentile, median levels of television viewing time were associated with higher K-10 scores (*B*[95% CI] = 0.16[0.02, 0.30]). Sex-stratified results are presented in Fig. 3. There were no clear differences between men and women for television time, occupational activity or household PA and distress associations. However, the effect estimates for transport PA as well as leisure PA and distress were larger for women. Including BMI as a confounder did not change these results (data not shown).

## Discussion

Our study contributes to the literature by investigating the dose–response associations between domain-specific PA, television viewing time and psychological distress, including sex-stratified models. We hypothesized that higher PA across domains and less television viewing time would be associated with reduced distress. We found higher leisure and transport PA associated with lower psychological distress, with this relationship greater among women. Undertaking some PA in the household domain was associated with lower distress; however, this relationship was non-linear, and the association moved towards null as household PA continued to increase. We found no association between occupational PA and distress. Higher television viewing time was associated with higher psychological distress.

Associations between PA and psychological distress differed across PA domains. Higher transport and leisure but not occupational PA were associated with less psychological distress. This finding replicated recent cross-sectional studies that demonstrated that the type, context and domain of PA influence the strength and direction of PA × mental health associations [11, 33–38]. We add to this literature through visual examination of the dose–response relationship between PA domains, television viewing time and distress, including sex-stratified models. Of note, less distress was evident with relatively low levels of transport (~ 10 MET h/wk) and leisure PA (~ 20 MET h/wk). This suggests that, for inactive people, improvement in mental health may not require large, unrealistic increases in transport or leisure PA. Transport and leisure PA may be beneficial when undertaken in natural outdoor environments due to stress reduction [39]. Additionally, these PA types may create an environment that allows distraction from stressful life events (e.g. walking to the train station after a stressful workday to unwind) [40].

We found that participants with median household PA had lower distress than those in the low quartile, although there was no difference compared with the most active quartile. However, a recent meta-analysis did not identify any relationship between household PA and mental health [11], whilst high occupational PA has been shown to associate with both good mental health and mental ill-health [11]. It is plausible that these types of PA do not facilitate the mental health benefits seen in leisure PA due to lack of intensity-driven output, skill mastery, self-efficacy and social support that are obtained by undertaking structured exercise, sports or other leisure activities with difficulty [41].

Another important factor is that employees with lower job grades have a higher proportion from socially disadvantaged and marginalized communities, who have less control and support at work, and engage in poorer health behaviours (e.g. lack of exercise, insufficient diet, smoking), with each of these factors alone being contributors of experiencing distress [42]. In fact, high levels of household or occupational PA may actually facilitate increased time spent in situations that are sources of stress [40]. Further, occupational and, to a lesser extent, household PA are generally obligatory, whereas leisure and transport PA can be considered optional. Although participation across all PA domains is recommended, people may experience substitutions from one domain to another that could have a resulting impact on their mental health (e.g. someone working as a construction worker with high occupational PA may experience musculoskeletal soreness from high exertion loads, leading to decreased participation in leisure PA) [43].

We found that more time spent watching television, a common sedentary behaviour, was associated with higher distress, which is similar to prior studies identifying the association between a sedentary lifestyle and worse mental health [33, 44]. The content of television viewed may also be relevant, with violent genres linked to anti-social and aggressive behaviour traits [45], and greater exposure to COVID-19 news media associated with greater psychological distress [46]. It is plausible that other sedentary behaviours that we did not investigate may contribute to increased risk of poor mental health, such as total sitting time or social media use [47]. Special consideration should be given to social media as a sedentary behaviour, as it is not simply total usage time that is an issue, but rather addictive and compulsive use that associates more strongly with poor mental health [47].

We found no clear differences between men and women for television viewing time, occupational or household PA and psychological distress associations. However, the effect estimates for transport and leisure PA and distress were larger for women. This finding is important given females have been shown to participate in lower recreational, transport and occupational PA then men, both for walking and vigorous-intensity PA [48]. Further, a pooled global study of 1.9 million participants found 23.4% of males had insufficient PA compared with 31.7% of females, with this discrepancy greater in high-income Western countries [49]. Thus, it is critical to promote PA in women, particularly transport and leisure PA, to bridge this gap and facilitate reductions in psychological distress that may be experienced by a higher proportion of physically active women.

There are numerous potential mechanisms for the relationship between PA and distress identified in our study, which may differ by the domain it is performed. Engaging in leisure PA such as structured exercise allows for a cascade of physiological adaptations that can contribute to improved physical health (e.g. cardiometabolic, musculoskeletal, neurobiological) and can reduce the risk of developing numerous chronic diseases [50]. Physical activity has been shown to stimulate several neuroplasticity processes related to depression, including increasing hippocampus and prefrontal volumes [51]. These benefits can improve self-efficacy, social support, confidence and quality of life, which contribute to improved mental health and reduced distress [50]; however, most of this research is conducted in leisure PA settings.

On the other hand, there may be numerous reasons for the link between low PA and high sedentary time with distress. Physical inactivity is a risk factor for numerous chronic diseases including some cancers, cardiovascular disease and metabolic conditions [52]. Living with multiple chronic conditions can exacerbate distress and reduce quality of life [53]. People living with poor physical or psychological health may experience difficulties in the ability to participate in leisure, transport, occupational or household PA due to lack of guidance, motivation, logistical or access issues. However, it could be argued these populations have the most to gain from adopting regular PA to improve their physical and psychological health.

There are several practical recommendations that can be applied to these data to promote behaviour change: (1) Given regular leisure PA may contribute to improved mental health, walking around the block, and utilizing social support such as a friend or family member may be a practical way to start. If time is a barrier, short PA bouts can be considered, particularly when commencing, which has recently been termed Snacktivity™ (e.g. squat whilst waiting for kettle to boil, have walking meetings) [54]. (2) Adults should explore opportunities to engage in transport PA such as walking or cycling to work or incorporating an active component of the journey such as getting on or off the bus 1–2 stops earlier. (3) Television viewing time should be broken up with consideration given to spending short bouts of time participating in leisure or household PA instead.

Our study has several key strengths including a large sample of Australian adults, examining multiple PA domains and television viewing time and examining sex-specific dose–response associations for these exposures with a mental health measure used widely for clinical and research use. Although our approach captures the relationship between domain-specific PA and psychological distress, most PA guidelines do not provide domain-specific recommendations [55], instead emphasizing quantities of PA regardless of context, making direct comparisons between our findings and public health recommendations challenging. Our results also have limitations and should be interpreted accordingly. By using a cross-sectional design, it becomes challenging to conclude whether distress is a cause or effect of PA participation across the domains, and as such, this issue could be alleviated using prospective studies. There were relatively few participants with poor mental health, with 12% likely to have psychological distress using K-10 cut-off scores. This limitation may have impaired our ability to detect significant associations between PA or television viewing time with distress, highlighting a need to investigate this association among vulnerable populations. The use of self-reported PA data is a limitation. Although the IPAQ-long has been validated extensively, moderately correlates with accelerometer data and is suggested for population-based studies [56], self-report data can allow for over- or under-reporting, which may attenuate risk estimates [57]. Whilst accelerometers can eliminate some bias, they do not differentiate between PA domains; thus, self-reported input is still required to answer this question. Although this study used a mental health questionnaire with clinical utility, it is not diagnostic and there may be a range of other factors (e.g. genetic, medical, socio-cultural, life events) that impact both mental health and domains of PA, and additionally our findings may only relate to chronic and not acute distress. The MCCS FUP2 data was collected in 2003–2007 [22], and leisure activities like on-demand streaming services have increased in use since then, which may affect the precise nature of sedentary time × mental health effect estimates. Finally, our investigation of occupational PA was limited to employed participants, which was around half the sample. As such, the generalizability of these findings to all mental health conditions cannot be assumed.

## Conclusion

Increasing PA and reducing television viewing time have been identified as important contributors to good mental health. Whilst our findings support this, they suggest that PA in the transport and leisure domains should be prioritized, particularly in women. The relationships for household PA and television viewing time were less clear, with no relationship between occupational PA and distress. These findings can assist in designing interventions to reduce psychological distress.

## Supplementary Information

Below is the link to the electronic supplementary material.

Supplementary file1 (DOCX 302 KB)

## Data Availability

The datasets generated during the current study are available via application from the corresponding author on reasonable request.
