# Supplementary material for: The Relationship Between Psychological Distress and Physical Activity Is Non-linear and Differs by Domain: a Cross-Sectional Study
Source: Int J Behav Med. 2022 Sep 30;30(5):673–81. doi: 10.1007/s12529-022-10130-5 (PMC9524734; doi:10.1007/s12529-022-10130-5)

**Supplement 1A: Directed acyclic graph for physical activity and TV time to mental health.** Confounders adjusted for in the final models include age, alcohol consumption, comorbidities, country of birth, education, marital status, SEIFA, sex, smoking, and working status. As physical activity prevents BMI increase, body composition is displayed as a mediator.


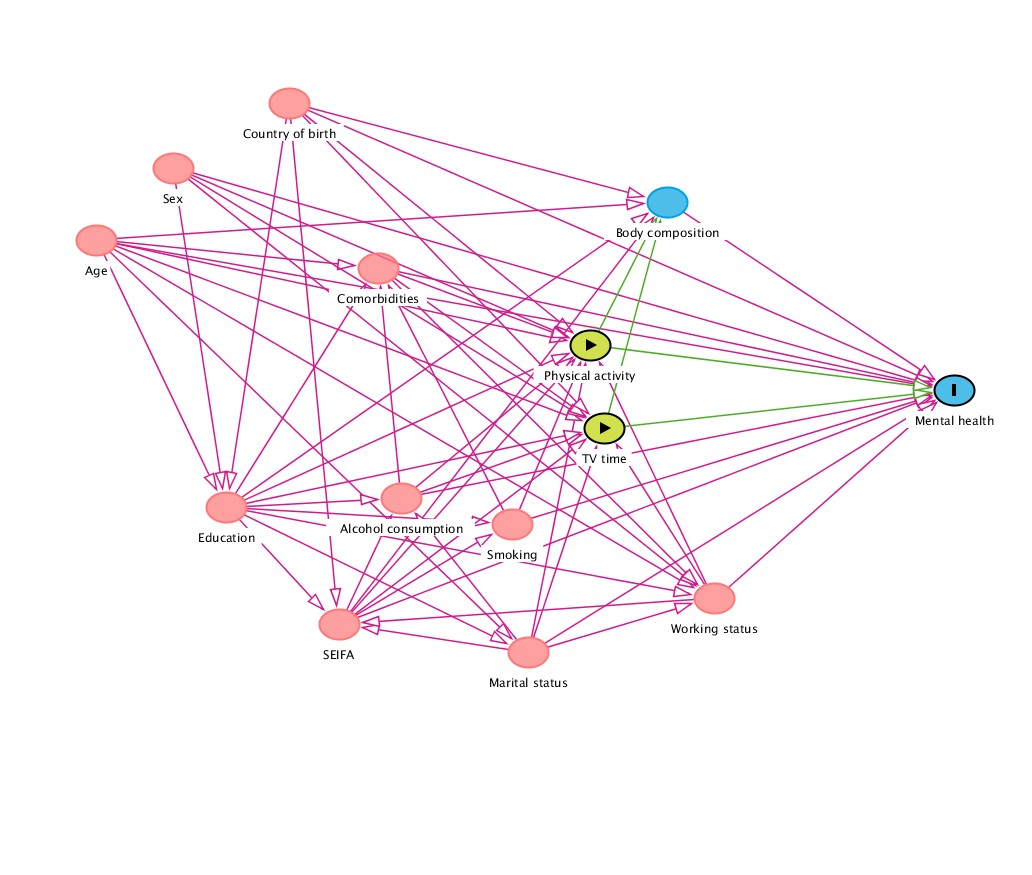


**Supplement 1B: Directed acyclic graph for physical activity and TV time to mental health.** Confounders adjusted for in the sensitivity analysis include age, alcohol consumption, body composition, comorbidities, country of birth, education, marital status, SEIFA, sex, smoking, and working status. As body composition has been identified as a correlate of physical activity, sedentary behaviour, and mental health, it is included as a confounder in sensitivity analyses.


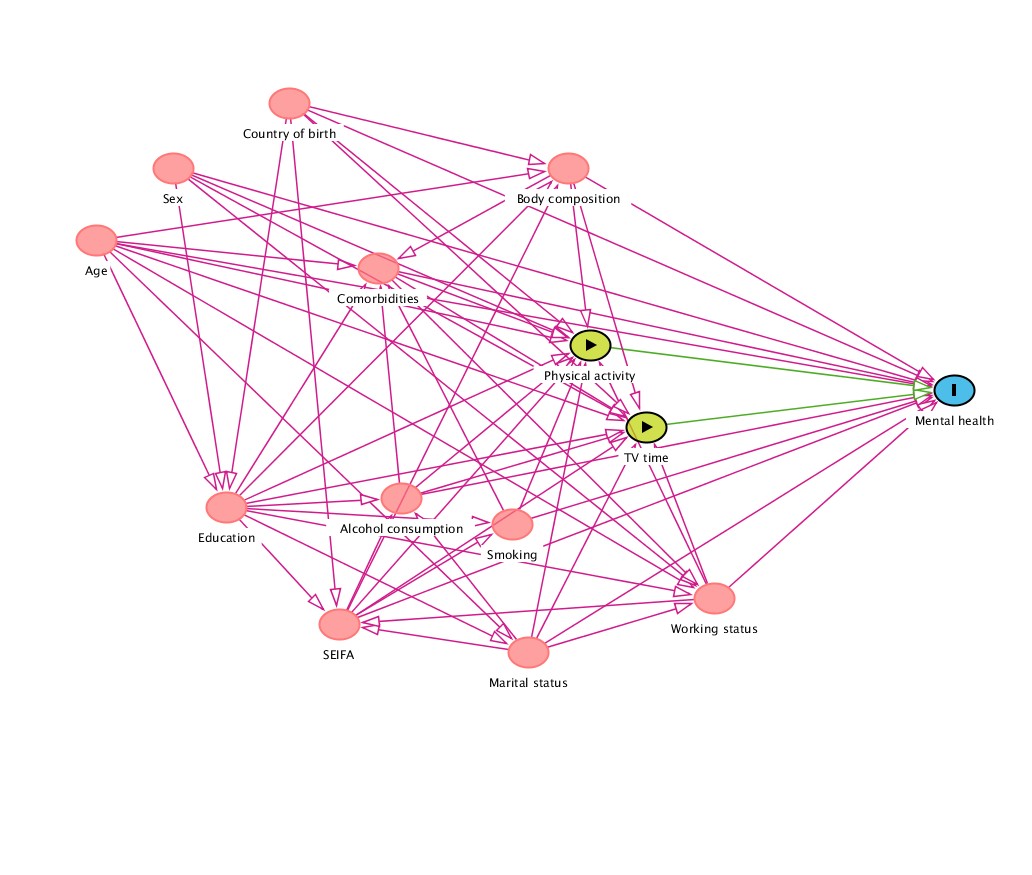

Supplement: Supplementary file 1 — Supplementary file1 (DOCX 302 KB) [file 12529_2022_10130_MOESM1_ESM.docx]
